# Feasibility and Efficacy of a Virtual Reality Social Prediction Training in Children and Young Adults with Congenital Cerebellar Malformations

**DOI:** 10.1007/s10803-024-06349-8

**Published:** 2024-05-03

**Authors:** Niccolò Butti, Emilia Biffi, Romina Romaniello, Alessandra Finisguerra, Enza Maria Valente, Sandra Strazzer, Renato Borgatti, Cosimo Urgesi

**Affiliations:** 1 Scientific Institute, IRCCS E. Medea, Bosisio Parini, Lecco, Italy; 2 IRCCS Mondino Foundation, Pavia, Italy; 3 Scientific Institute, IRCCS E. Medea, Pasian di Prato, Udine, Italy; 4 Department of Molecular Medicine, University of Pavia, Pavia, Italy; 5 Department of Brain and Behavioral Sciences, University of Pavia, Pavia, Italy; 6 Laboratory of Cognitive Neuroscience, Department of Languages and Literatures, Communication, Education and Society, University of Udine, Udine, Italy

**Keywords:** Social prediction, Cerebellum, Virtual reality, Rehabilitation, Theory of mind, Cerebellar cognitive affective syndrome—CCAS

## Abstract

This study tested the feasibility and efficacy of a Virtual Reality (VR) social prediction training (VR-Spirit) specifically designed for patients with congenital cerebellar malformation. The study is a randomised controlled trial in which 28 cerebellar patients aged 7–25 yo were randomly allocated to the VR-Spirit or to a control intervention in VR. The VR-Spirit required participants to compete with different avatars in scenarios that prompted them to form predictions about avatars’ intentions. The control intervention consisted of games currently adopted for motor rehabilitation. Social prediction as well as secondary neuropsychological and behavioural outcomes were assessed at the beginning (T0), at the end (T2) and after 2 months (T3). The experimental group showed a significant increase, compared to the control participants, in social prediction assessed through a VR task. Moreover, at least at T3, the VR-Spirit enhanced the use of contextual predictions in a computer-based action prediction task. Importantly, these effects were generalized to secondary neuropsychological outcomes, specifically theory of mind and, only at T2, inhibition. No differences between the interventions were detected on emotional-behavioural problems. Lastly, both interventions showed high feasibility and acceptability. These findings confirm that it is possible to develop condition-specific rehabilitative training on the basis of neurocognitive functions impaired in case of congenital malformation. The VR-Spirit demonstrated to generalize its effects to theory of mind abilities, and it might be thus extended to other neurodevelopmental disorders that present social perception deficits and alterations of predictive processing.

**Trial registration**: ISRCTN, ID: ISRCTN22332873. Retrospectively registered on 12 March 2018.

**Supplementary Information:**

The online version contains supplementary material available at 10.1007/s10803-024-06349-8.

## Introduction

There is extensive evidence that cerebellar alterations result not only in motor impairments, but also in a complex constellation of neuropsychological deficits and abnormalities in behaviour and affect regulation, referred to as the Cerebellar Cognitive Affective Syndrome (CCAS) (Manto & Mariën, 2015; Schmahmann & Sherman, 1998; Tavano et al., 2007). Such a variety of cognitive, social, and behavioural symptoms, characterized by an augmented or diminished response to external and internal stimuli (Schmahmann, 2004), is thought to depend on the prominent role of the cerebellum in predictive processing (Schmahmann, 2019; Sokolov et al., 2017). Through its main functions of providing predictive internal models and error-signalling (Wolpert et al., 1998), the cerebellum would act as a general regulator and monitor of the activity of different brain networks, from sensorimotor processing to language, regardless of the type of information being fed (Peterburs & Desmond, 2016; Siman-Tov et al., 2019). In detail, the cerebellum would extract contextual regularities and provide forward models to cortical networks, with the ultimate outcome to allow overcoming uncertainty and selecting the best matching between external information and internal expectations (Ishikawa et al., 2016; Leggio & Molinari, 2015).

This predictive function might be particularly critical in social contexts, which are inherently dynamic and require continuous adaptation of one’s expectations about others’ mental states and behaviours (Brown & Brüne, 2012; Stoodley & Tsai, 2021; Van Overwalle et al., 2019a, 2019b). Notably, recent evidence has documented that alterations of cerebellar functions, either in clinical conditions or after non-invasive brain stimulation, affect the processing of social stimuli more than the processing of inanimate objects (Cattaneo et al., 2012; Ma et al., 2021; Oldrati et al., 2021; Van Overwalle et al., 2019). Evidence of structural loops between the lateral posterior cerebellum and the superior temporal sulcus suggests that the cerebellum contributes to understanding biological movements (Sokolov et al., 2010, 2014). The vermis is linked with limbic areas and circuits involved in affect processing and regulation (Adamaszek et al., 2017). The reciprocal connections between the posterior cerebellum and cortical areas engaged by the so-called mentalizing network (Van Overwalle & Mariën, 2016; Van Overwalle et al., 2020) suggest that the cerebellum might apply its predictive computation also on complex social information. Accordingly, an increasing number of studies have investigated social perception in cerebellar patients, reporting deficits at multiple levels of social processing, from understanding biological movement (Abdelgabar et al., 2019) to affect recognition (Adamaszek et al., 2015; Hoche et al., 2016) and theory of mind abilities (Clausi et al., 2019; Sokolovsky et al., 2010). Structural and functional abnormalities of the cerebellum have been documented in clinical populations characterised by social impairments, such as Autism Spectrum Disorder (ASD) (Olivito et al., 2017; Van Overwalle et al., 2020). Early injuries and congenital malformations of the cerebellum can interfere also with the development of the connected cortical nodes of social cognition through mechanisms of developmental diaschisis (Stoodley & Limperopoulos, 2016).

Notably, research on rehabilitative interventions to treat CCAS is scarce (Argyropoulos et al., 2020; Gagliardi et al., 2015; Ito et al., 2010; Ruffieux et al., 2017). To the best of our knowledge, literature does not report interventions specifically designed to target social perception deficits in cerebellar patients. To fill this gap, the Virtual Reality Social Prediction Improvement and Rehabilitation Intensive Training (VR-Spirit) was developed. This intensive training program delivered in virtual reality (VR) aims to enhance social prediction in children, adolescents, and young adults with congenital cerebellar malformations (CCM). Briefly, this training exploits a design based on probabilistic learning of social events in motivating and child-friendly environments. During the training, patients are asked to reach a desired target; importantly, to achieve success in their actions, they must predict the behaviour of other competing virtual avatars. To verify the efficacy and specificity of the VR-Spirit, as well as its feasibility and acceptability, the effects of the VR-Spirit on outcome measures of social prediction ability and neuropsychological functions were compared to the effects induced by a VR motor rehabilitation training, in a randomised controlled trial. The study protocol was previously registered (ISRCTN 22332873) and published (Butti et al., 2020a). A preliminary paper has reported validating evidence on the social effects of the VR-Spirit, highlighting short-term improvements on social prediction (Urgesi et al., 2021). Here, after trial conclusion, we reported the results on the efficacy and specificity of the VR-Spirit on social prediction and its generalisation to secondary neuropsychological outcomes, particularly social perception skills, as well as its feasibility and acceptability. In particular, we hypothesized that the VR-Spirit, compared to the control VR-based motor training, should: (1) enhance social prediction ability resulting in better understanding of other people’s intentions and behaviours; (2) improve cognitive performance in specific domains, particularly in social perception; and (3) be feasible and well tolerated in children, adolescents and young adults with CCM.

## Methods

### Trial Design

The study applied a single-centre, randomised, active controlled trial design, in which patients were allocated to the VR-Spirit or to the control VR-assisted motor rehabilitation intervention. A stratified permuted block randomisation procedure was used for allocation, considering age and cognitive level (i.e., the most recently available Full-Scale Intelligent Quotient, FSIQ), as stratification factors. Participants were assigned to one of six blocks according to the two stratification variables, considering two levels of age (7.0–12.9, ≥ 13.0 yo) and three levels of cognitive functioning (46–60, 61–80, > 80 FSIQ). A sample size of 42 participants was estimated through an a-priori power analysis to detect a large (*d* = 0.8) between-group difference (independent sample *t* test, two-tailed) with a power of 0.80 and an alpha level set at 0.05 (Butti et al., 2020a). Thus, it was planned to assign up to eight patients per block to achieve and overcome the pre-determined sample size. Within each block, participants were allocated to the VR-Spirit or the active control group on the basis of pre-established permuted sequences, which were generated before enrolling the first participant. Full details of the trial design and randomization procedure have been previously registered and published (Butti et al., 2020a).

### Participants

Patients were enrolled at the Child Neuropsychiatry and Neurorehabilitation Unit of the Scientific Institute, IRCCS E. Medea (Bosisio Parini, Italy), to which they were referred for routine medical and rehabilitative checks. Inclusion criteria were: (i) diagnosis of non-progressive congenital cerebellar malformation; (ii) age ranging from 7 to 25 yo; (iii) absence of severe cognitive impairment (FSIQ > 45), as shown by the most recent (< 6 months) available FSIQ at the age-corresponding Wechsler scale; iv) absence of severe motor and visual impairments that could interfere with task execution; (v) not being simultaneously involved or having been involved in the previous 6 months in any formal VR intervention study. To broaden the generalizability of the training, we included both participants in developmental age and young adults, so that the rehabilitative training could take advantage of still ongoing neurodevelopmental processes and of the related plasticity (de Lange et al., 2017). Accordingly, regional cerebellar volumes were found to be differently associated with sensorimotor and cognitive task performance in young and older adults (Bernard & Seidler, 2013). Please note that criterion iii was initially FSIQ > 60 and it was modified in December 2018 in order to include patients with moderate cognitive delay (45 < FSIQ ≤ 55). This choice was made since preliminary outcomes indicated good feasibility and acceptability of the trial independently by cognitive level. The repetitiveness of the task, the constant presence of a therapist to remember instructions, and the ease of movement in the virtual environment likely helped participants understand and perform the training. However, FSIQ > 45 was maintained as an inclusion criterion to ensure patients’ adherence to the training and testing procedure. For three participants, the FSIQ was not available due to severe linguistic impairments, so that the IQ was calculated from the perceptual reasoning index at the age-corresponding Wechsler scale.

The first participant was enrolled in February 2018 and the last one in September 2021, with the last follow-up assessment completed in December 2021. The trial was suspended from March to July 2020 due to the Covid-19 pandemic. Although the trial was later resumed and the end date was postponed to January 2022, only five participants were enrolled after July 2020 due to pandemic-related restrictions and safety procedures. These unforeseen events prevented the target sample size of 42 participants from being reached. This target sample size was estimated on the basis of the minimal clinically relevant change expected on the primary outcome measure (for details see Butti et al., 2020a).

**Fig. 1:**
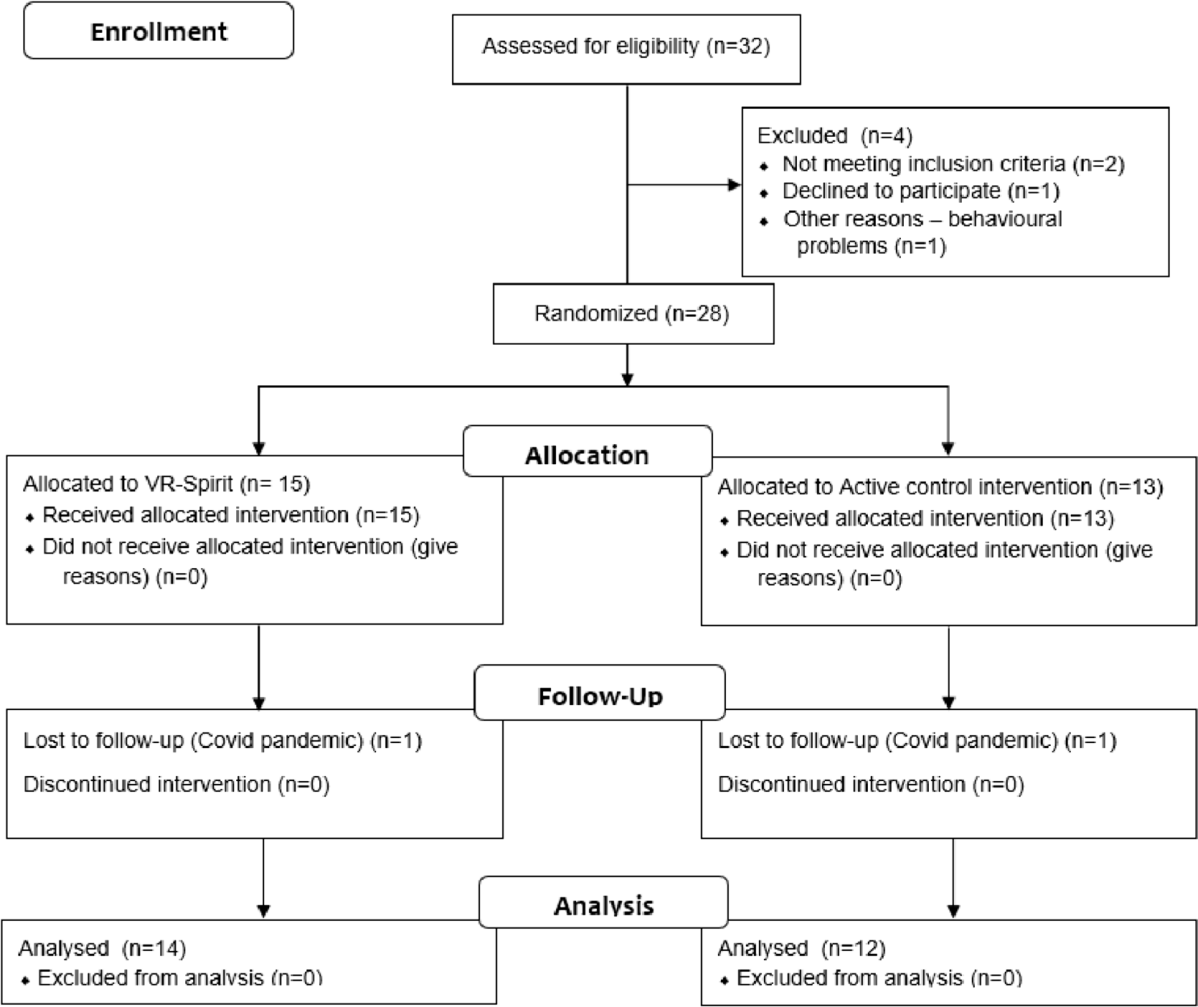
CONSORT flow diagram of recruitment

An administrative staff member, blind to the trial design, enrolled the 32 participants and organized hospitalisation according to each patient and family’s needs and availability. When further screened for eligibility, three patients were excluded from allocation since they did not meet eligibility criteria. In detail, for one patient a diagnosis of progressive ataxia was formulated; for one, the severity of motor impairments prevented task execution; and a third was dismissed due to severe behavioural problems. Moreover, a family declined to participate due to time constraints. A total of 28 patients (21 M; mean age = 13.1 yo) were then assigned to the experimental or control group according to the allocation ratio. Two participants (one per group) did not complete the follow-up assessment due to Covid-19 pandemic, so that data of 26 participants were considered for the follow-up effect (Fig. 1).

A sensitivity analysis conducted with G*Power 3.1 (Faul et al., 2007) showed that the achieved sample size allowed us to detect large effect sizes of at least *d* = 1.1, at T2, and of at least *d* = 1.15, at follow up (T3), with a power of 0.8 and an alpha level set at 0.05.

As our sample did not reach the targeted size, the six blocks of stratified randomisation were unevenly filled (see supplementary Table 1). However, the two groups were balanced in terms of biological sex and stratification variables (i.e., age, FSIQ), confirming successful randomization (Table 1).

**Table 1:** Demographic and clinical information for the two groups

	VR-Spirit	Active control group	Statistical comparison	
			χ^*2*^*/t*	*p* value
Demographic information				
Biological sex—M:F	11:4	10:3	0.05	0.82
Age—Mean (SD)	13.4 (3.8)	12.8 (5.2)	−0.34	0.74
Clinical information				
IQ—Mean (SD)	72 (26)	67 (21)	−0.56	0.58
Primary cerebellar malformation (N)	Hypoplasia (5), Molar tooth (3), Vermis hypoplasia (5), Rhomboencephalosynapsis (1), Cerebellar folia dysplasia (1)	Hypoplasia (2), Molar tooth (8), Vermis hypoplasia (3)		
Syndromic/genetic diagnosis (N)	Joubert syndrome (3), ITPR1 gene mutation (1), unknown (11)	Joubert syndrome (8), unknown (5)		

Most participants followed a differentiated and/or reduced school programme and were assisted by a special education teacher in accordance with Italian laws (for detailed information of each participant see supplementary tables 2 and 3).

The procedures of this study were approved by the local Ethics Committee and agreed with the principles expressed in the Declaration of Helsinki. Patients and their parents were asked to sign a written informed consent form before starting any procedures.

### Interventions

Both the VR-Spirit and control rehabilitation trainings were administered in the Gait Real-time Analysis Interactive Laboratory (GRAIL, Motek, Amsterdam, NL) of the Scientific Institute Medea. The GRAIL system is an integrated platform equipped with a treadmill, an integrated motion-capture system, and a 180° cylindrical projection screen on which the VR scenarios are projected. Both interventions consisted of eight 45-min daily sessions administered over 2 weeks by a GRAIL-patented physical therapist. The therapist placed two reflective markers on the posterior superior iliac spines, which traced patient’s movements in the virtual environment. Namely, participants had to move forward or backward to go faster or slower, respectively; they had to shift the pelvis to turn right or left. This movement system limited the impact of gait problems (e.g., ataxia).

The VR-Spirit presented participants with a playground scenario in which they were asked to compete with one of four avatars in reaching one of three pieces of recreational equipment (a swing, a circular carousel and a rocking carousel). Two avatars were male adolescents and two were female adolescents; each individual avatar was clearly identifiable by hair and t-shirt colours. For each trial of the training, one of the avatars was visibly positioned next to the patient; the avatar then started moving along a 9-m straight-line path and turned into one of the three 3-m long branches directed to one of the recreational pieces. Each avatar’s choices of the recreational objects were biased towards pre-established probabilistic associations. In detail, three avatars preferred a specific object in 80% of the trials and moved towards each of the other two objects in 10% of the trials. One avatar moved in a pseudo-random way, according to avatar-object probabilistic associations set at 33%. Each trial lasted 15 s and ended when the avatar reached the chosen object. Participants’ velocity was set so that they could pass the avatar only before the crossroad; thereafter the velocity of the participants’ movements in the VR-scenario was limited to 2 m/s, being slower than the avatars’ movements. This way, participants were forced to anticipate avatars’ movements without any perceptual clues about their direction, thus prompting them to form predictive models of the avatar-object associations. Namely, participants had to predict the location where the avatar was walking in order to get to the chosen recreational equipment before him/her. When participants anticipated the avatar in reaching the chosen object, they scored a point in the game and obtained auditory (clapping sound) and visual (activation of the object) reinforcements. The object reached by the avatar was always visible to the participant, in order to provide information on the avatar’s preferences also for unsuccessful trials.

Considering 20 trials per avatar, each session consisted of 80 trials in which the pre-established events were presented in a random order. Moreover, four different versions were created according to a counterbalance of the avatar-object associations. Administration of these sessions followed the same order in the 2 weeks but was randomised and counterbalanced between participants. At the end of each session, lasting around 30 min, participants were presented with one of four motor games selected from the GRAIL kit, which were also adopted in the active control training.

Participants assigned to the active control group played a custom-made navigational game, in which they had to conduct a ball out of three mazes, and all the four GRAIL games designed for motor rehabilitation. All games were selected for the absence of social interactions and did not require any form of prediction abilities. The order of administration of these games was kept constant across sessions but counterbalanced between participants (for further details see Butti et al., 2020a).

**Fig. 2:**
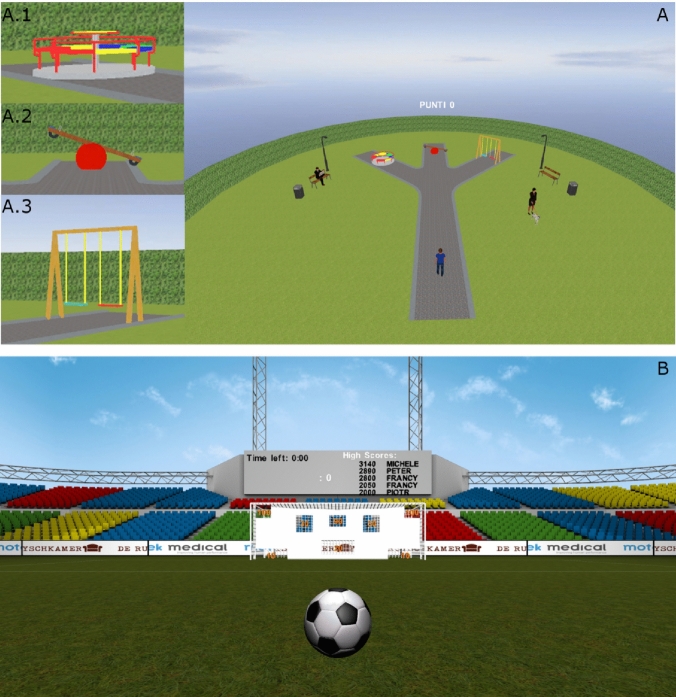
The training scenario and a control motor game. **A** VR-SPIRIT intervention: the playground scenario with a circular carousel (A.1), a rocking carousel (A.2) and a swing (A.3) at the end of the path. **B** One of the games included in the active control intervention: the soccer game

The training scenario and an example picture of a game from the control motor intervention are reported in Fig. 2.

### Assessment and Outcome Measures

All participants underwent assessment of different primary and secondary outcomes at three different time points: before the training (T0), at the end of the training (T2), in a follow-up session 2 months after the end of the training (T3).

Social prediction was set as the primary outcome and assessed through two different paradigms. First, a VR social prediction task was administered at T0 and T2. This task mirrored exactly the design of the VR-Spirit, but adopted a different scenario, namely a sweet stands’ environment, and different avatars. This way it was possible to assess improvements of social prediction independently from practice effects. Please note that before starting the first evaluation session, participants were administered a short and effective navigational training to learn how to move within the GRAIL VR environments. In order to avoid repetition of the same events between T0 and T2, two different versions of the task were created by changing the avatar-object associations and their administration was counterbalanced between patients of the same group. A prediction score (VR-prediction score) was computed by assigning one point every time the participant moved according to the avatars’ preferred choice (i.e., the 80% avatar-object association). Namely, when the participants reached the object preferred by the avatars before him/her, we considered the trial as a correct prediction. Conversely, a wrong prediction corresponded to the choice of one of the two seldom preferred sweet stands (i.e., the 10% avatar-object association). The trials with the avatar moving in a pseudo-random way (i.e., the 33% avatar-object association) were not considered in the VR-prediction score. This measure ensured ruling out noise due to the use of random strategies, and it was found to be sensitive to changes across time (Urgesi et al., 2021).

Then, social prediction was also assessed at T0, T2 and T3 through a validated, computer-based action prediction task previously adopted in paediatric clinical populations (Amoruso et al., 2019; Butti et al.,2020b). This task consisted of short videos displaying an actor preforming reaching-to-grasp movements towards one of two possible objects (i.e., a glass or an apple) with the intention to drink/eat it or to offer it. The actions were embedded in a scenario containing different contextual cues, namely, coloured tablecloths or coloured plates. Participants were required to recognise which was actor’s intention (i.e., to drink/eat vs. to offer). In the familiarization phase, videos stopped when the actor’s hand reached the object, so that participants could rely on full kinematics information to understand the action unfolding. Crucially, in this phase, the association between contextual cues and actor’s intentions was implicitly biased with pre-established probability of co-occurrence (10%, 40%, 60%, 90%), in order to prompt the formation of contextual predictive models (i.e., priors). During the testing phase, the same videos were presented but occluded well before the hand-object contact, so that participants should rely on the contextual priors to overcome kinematics ambiguity and to understand actor’s intention. For each participant, a standardised beta coefficient (herein after beta index) was calculated across the trials using a regression analysis with probability and accuracy as the independent and dependent variables, respectively. This index is a proxy of the steepness of the regression line between the four levels of probability, and it thus represents the strength of contextual prior use. Importantly, the beta index was found to be associated with social perception skills in cerebellar patients (Butti et al., 2020b; Urgesi et al., 2021). Accordingly, an increase of moderate effect size in the beta index (0.13 mean change) was considered as a clinically relevant improvement in social prediction, with likely effect on social perception (Butti et al., 2020a). The accuracy in the familiarization phase of the same action prediction task was considered as a secondary outcome measure of implicit learning.

Moreover, selected subtests of the Italian version of the NEPSY-II testing battery (Korkman et al., 2007) were administered across the three time points (i.e., T0, T2, T3) to assess different secondary neuropsychological outcomes. The NEPSY-II is one of the most world-wide adopted batteries for neuropsychological testing in developmental age, and it has been used to investigate the neuropsychological functioning of different clinical populations (Ferrari et al., 2022; Narzisi et al., 2013; Rasmussen et al., 2013). In detail, the following subtests were administered: theory of mind and affect recognition in the social perception domain; visual attention and inhibition in the attention and executive functions domain; picture and geometric puzzles for visuospatial processing; memory for designs for visuospatial memory; fingertip tapping for sensorimotor functions. Raw scores of each subtest were transformed into scaled score (mean = 10, SD = 3) according to the normative standardization tables (Urgesi et al., 2011). Average scaled scores (composite score) were calculated for the subtests providing multiple scaled scores for the same outcome, namely: naming, inhibition and switching for inhibition; immediate and delayed recall, for memory for designs; picture and geometric puzzles, for visuospatial processing.

Lastly, parents were asked to fill the Child Behavior Checklist (CBCL) 6–18 before the training (T0) and at the follow-up session (T3). The CBCL is a widely-adopted questionnaire regarding the presence of emotional-behavioural problems in children and adolescents (Achenbach, 2011; Frigerio et al., 2004), providing standardized T-scores (mean = 50, SD = 10) on eight syndromic scales. Two participants were over 18 years and thus they were not administered the questionnaire, while parents of one participant assigned to the experimental group did not fulfil the questionnaire at the follow-up assessment.

**Fig. 3:**
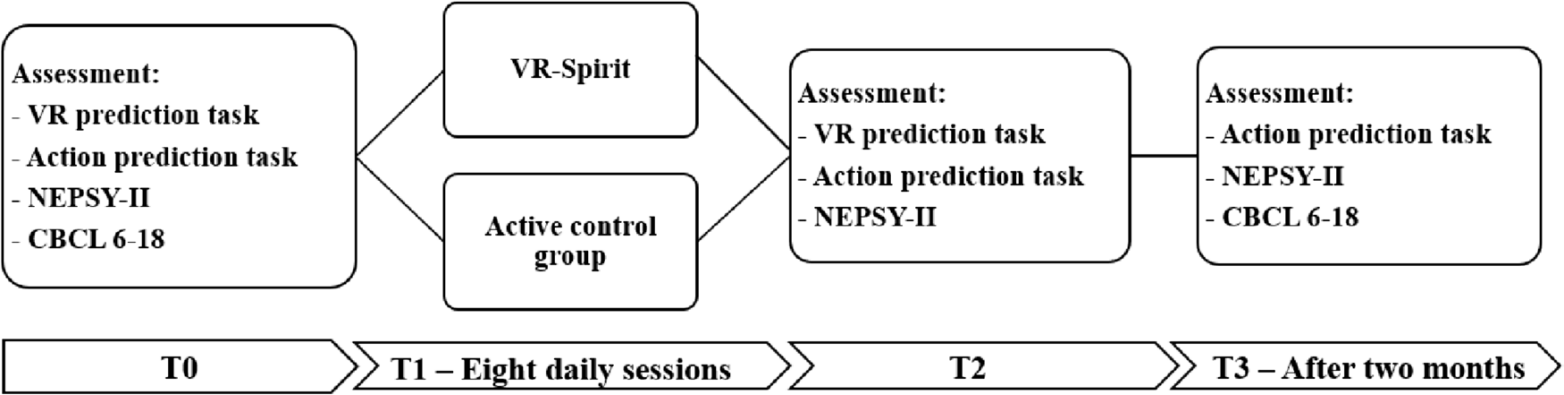
Timeline of the trial and assessments

A psychologist with expertise in neuropsychological testing, who was not blind to allocation, was responsible for all data collection. A resume of the assessments at the different timepoints is reported in Fig. 3.

### Feasibility and Acceptability

Feasibility and acceptability of the VR-Spirit for the target population were assessed. For the former aspect, the number of dropouts, that is the number of participants who declined to complete the training, and the number of sessions completed for each participant were collected. To assess acceptability, self-report and parent-report questionnaires were administered at the end of either training (T2). The questionnaires were adapted from a previous study on cognitive rehabilitation in paediatric patients with neurological disorders (Corti et al., 2018). The self-report questionnaire assessed recommendation level, required effort, pleasantness, comprehensibility of the intervention, difficulties to start and to continue the training. The parent-report version assessed recommendation level and satisfaction, as well as perception of their child’s required effort, difficulties to start and to continue the training. Answers were collected on a five-point Likert-like scale, from “not at all” = 1 to “a lot” = 5. The same Likert scale was used to assess negative (e.g., difficulties to start and continue the training) and positive (e.g., pleasantness, satisfaction) features of the training, thus limiting the impact of a likely tendency to affirmative answers in the self-report questionnaire due to the cognitive impairments.

### Data Handling and Statistical Analysis

According to study protocol, all participants who had completed the pre- and post-treatment evaluation sessions were considered in the outcome analysis. Given the limited sample size and observation points, no missing data was imputed (Dziura et al., 2013). Since two patients did not participate in the follow-up session, the analysis on the follow-up effect were performed on 26 participants. Primary and secondary outcomes of the two patient groups were inspected through descriptive statistics, and independent sample *t* tests were used to verify group differences at baseline (T0). For each outcome measure, the arithmetic differences between T2 and T0 (herein after *training effect delta*) as well as between T3 and T0 (hereinafter *follow-up effect delta*) were calculated (with higher values corresponding to stronger effects). For the primary outcome measure, the delta values of the two groups were compared by means of independent sample *t* tests, adopting the Bonferroni correction to control for multiple comparisons. For the action prediction task, these delta measures were also inspected individually to check whether and how many patients in each group showed an increase equal to or greater than the pre-determined minimal clinically important change (0.13). Moreover, to further investigate between-group differences after the training, exploratory *t* tests were conducted on the beta index values at T2 and T3.

As concerns the secondary outcomes, independent sample *t* tests were executed using the delta values of accuracy in the action prediction familiarization phase as dependent variable, while separate multivariate analysis of variance (MANOVA) models were run for the neuropsychological and the behavioural outcomes, separately for the training effect delta and for the follow-up effect delta, with group as categorical factor. In detail, for the neuropsychological outcomes, the delta measures of theory of mind, affect recognition, visual attention, inhibition (composite score), memory for designs (composite score), visuospatial processing (composite score) and fingertip tapping, were entered as dependent variables. Since three participants were not able to execute the inhibition and fingertip tapping subtests due to linguistic and fine-motor impairments, consequent MANOVAs were performed excluding these subtests. For the behavioural outcomes, we inserted as dependent variables in a MANOVA design, with group as a categorical factor, the follow-up delta score of the T-scores obtained at the syndromic scales of the CBCL 6–18, namely anxiety/depressive symptoms, social withdrawal, somatic complaints, social problems, thought problems, attention problems, rule-breaking behaviour. and aggressive behaviour. For each MANOVA model, univariate results were also calculated to observe differences in specific outcomes. Lastly, each answer at the acceptability questionnaires was compared between groups through Mann–Whitney *U* tests.

All analyses were performed with the Statistica software version 8.0 (Statsoft, Tulsa, OK). Effect sizes were reported with their 95% confidence interval (CI). Significance threshold was set at *p* = 0.05 for all statistical tests. Effect sizes were estimated and reported as Cohen’s *d* for pairwise comparisons, adopting conventional cut-off of 0.2, 0.5, and 0.8 for small, medium, and large effect sizes, respectively, and as partial eta squared (*n*^*2*^_*p*_) for ANOVA designs, adopting conventional cut-off of 0.01, 0.06, and 0.14 for small, medium, and large effect sizes, respectively (Cohen, 2013).

## Results

Descriptive statistics for the primary and secondary outcomes at the three timepoints are reported in Table 2.

**Table 2:** Descriptive statistics (mean and SD) for the primary and secondary outcomes at the three timepoints in the two groups

	VR-Spirit			Active control group		
	T0	T2	T3	T0	T2	T3
Primary outcome measure						
Social prediction						
VR-Prediction score	32.9 (8.4)	51.3 (5.2)		34.4 (10.5)	37.5 (8.1)	
Beta index	−0.03 (0.71)	0.25 (0.51)	0.26 (0.52)	−0.08 (0.53)	−0.05 (0.59)	−0.21 (0.54)
Secondary outcome measure						
Implicit learning						
Action prediction familiarization phase—accuracy	93%	94%	93%	91%	91%	92%
NEPSY-II scaled scores						
Social perception						
Theory of mind	4.7 (3.8)	7.3 (3.8)	7.7 (4.3)	6.2 (4.5)	6.8 (3.9)	5.5 (4.3)
Affect recognition	5.8 (4.1)	7.3 (4.5)	7.6 (3.6)	5.4 (3.1)	6.5 (3.3)	5.5 (3.6)
Visual attention						
Visual attention	4.3 (4.0)	5.7 (5.1)	7.0 (5.6)	4.5 (3.7)	6.3 (4.0)	5.8 (4.8)
Executive functions						
Inhibition	5.6 (3.7)	7.9 (3.4)	8.7 (3.6)	4.8 (2.4)	5.8 (3.3)	6.3 (3.4)
Visuospatial processing						
Picture and geometric puzzles	5.5 (4.2)	7.0 (4.9)	7.7 (5.2)	5.6 (3.2)	7.3 (4.1)	7.3 (4.2)
Visuospatial memory						
Memory for drawings	3.4 (3.7)	6.0 (4.8)	6.4 (5.1)	3.5 (3.9)	6.6 (4.6)	6.5 (4.6)
Sensorimotor functions						
Fingertip tapping	8.2 (4.6)	8.5 (4.7)	9.4 (4.5)	8.5 (3.5)	8.3 (3.5)	8.7 (3.5)
CBCL T-scores						
Emotional-behavioural problems						
Anxious/Depressed	60 (6)		57 (8)	59 (10)		59 (8)
Social withdrawal	58 (9)		61 (11)	58 (7)		60 (7)
Somatic complaints	59 (9)		56 (8)	58 (8)		57 (7)
Social problems	63 (10)		61 (9)	63 (6)		63 (7)
Thought problems	59 (10)		57 (10)	59 (10)		57 (8)
Attention problems	60 (11)		60 (10)	60 (5)		60 (6)
Rule-breaking behaviours	55 (7)		55 (7)	58 (9)		60 (8)
Aggressive behaviours	57 (9)		54 (7)	54 (18)		60 (11)

The analyses indicated that the two groups were comparable on all assessed outcomes at baseline (all *t* < 0.98, all *p* > 0.33).

### Primary Outcomes

The analysis revealed a significantly greater increase of the VR prediction score in the experimental compared to the control group, with large effect size (*t*_*26*_ =  − 4.85; *p* < 0.001; Cohen’s *d* = 1.84, 95% CI 0.91–2.66).

**Fig. 4:**
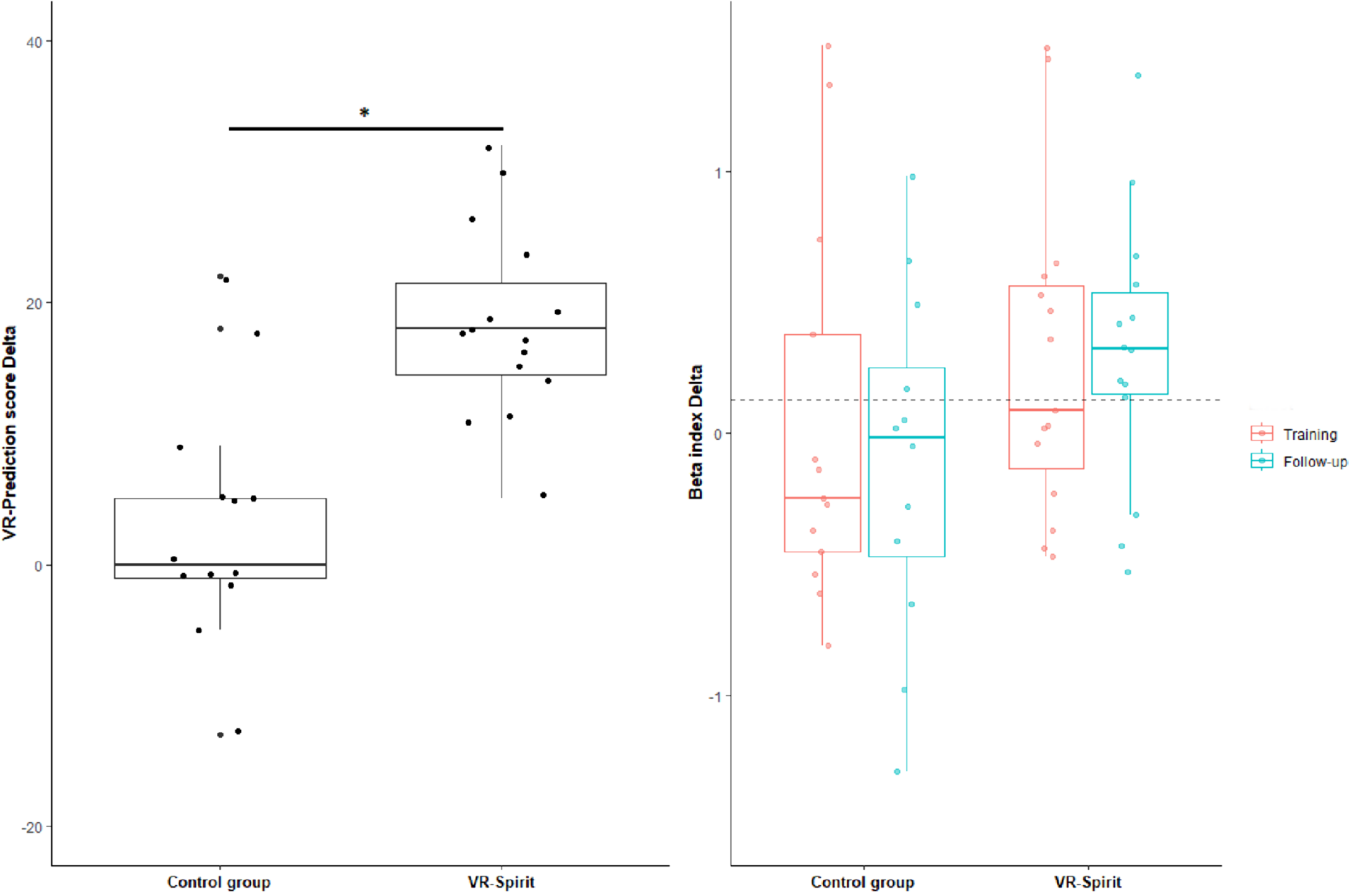
Boxplot of the training effect delta on the VR-prediction score and training and follow-up effect delta on the beta index in the two groups. The dotted grey line represents the estimated minimal significant improvement on the beta index. Asterisk indicates significant comparison

The between-group difference at the beta index for the training effect delta was small to medium and non-significant (*t*_*26*_ = 0.96; *p* = 0.344; Cohen’s *d* = 0.37, 95% CI − 0.39–1.10), while a medium-to-large effect of greater follow-up delta in the VR-Spirit than in the control group was marginally significant (*t*_*24*_ = 1.80; *p* = 0.083; Cohen’s *d* = 0.71, 95% CI − 0.11–1.48). At individual level, only four participants of the control group showed an increase above the clinically significant value (0.13), whereas, for the VR-Spirit group, 7 and 11 patients presented such an improvement at T2 and T3, respectively. Results on the primary outcome measures are reported in Fig. 4.

**Fig. 5:**
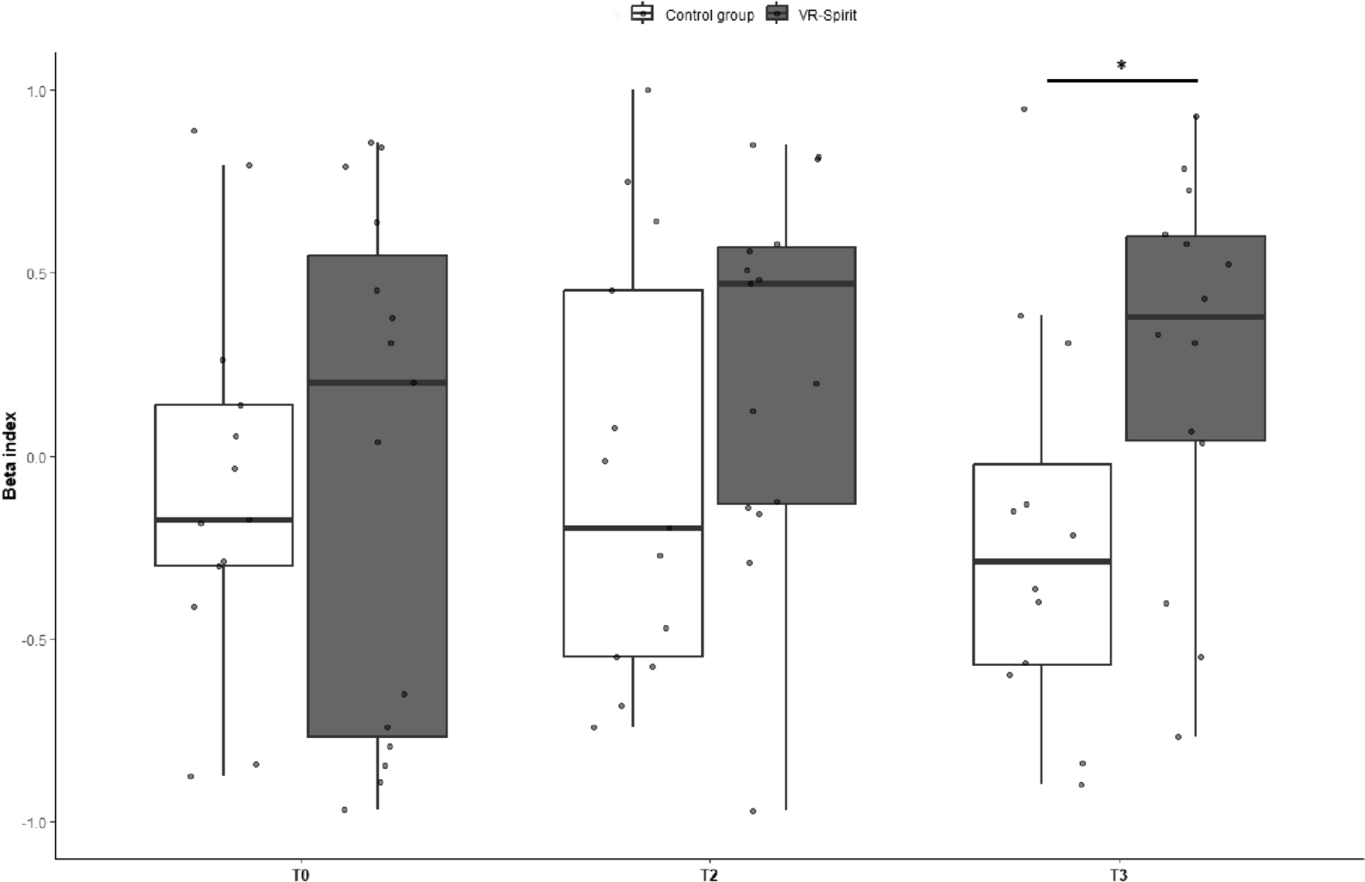
Boxplot of the beta index at the three time points in the two groups. Asterisk indicates significant comparison

Exploratory *t* tests confirmed that the between-group difference in the beta index at T2 was medium and non-significant (*t*_*26*_ = 1.41; *p* = 0.169; Cohen’s *d* = 0.54, 95% CI − 0.23–1.28), but at T3 the beta was greater in the VR-Spirit than in the control group, with a large effect size (*t*_*24*_ = 2.25; *p* = 0.034; Cohen’s *d* = 0.88, 95% CI 0.05–1.66) (Fig. 5).

### Secondary Outcomes

No differences were detected on delta accuracy in the action prediction familiarization phase (all *t* <|0.62|; all *p* > 0.54), indicating that the VR-Spirit did not affect action perception in general and the two groups were similarly exposed to the embedded probabilistic context-action associations. Thus, the VR-Spirit effects on the testing phase (our primary outcome measure) were due to an enhanced use of previously learned contextual expectations.

As concerns the neuropsychological outcomes, the two groups were overall comparable on the training effect delta (*F*_7,17_ = 1.83; *p* = 0.145; *n*^*2*^_*p*_ = 0.43, 95% CI 0.00–0.50), indicating that both groups increased their general performance at the NEPSY-II. However, significant differences were detected for the inhibition (*F*_1,23_ = 4.35; *p* = 0.048; *n*^*2*^_*p*_ = 0.16, 95% CI 0.00–0.40) and, marginally, for the theory of mind (*F*_1,23_ = 4.27; *p* = 0.05; *n*^*2*^_*p*_ = 0.16, 95% CI 0.00–0.40) subtests, both indicating higher increases after the VR-Spirit compared to the active control training. All other comparisons were non-significant (all *F* < 1.53; all *p* > 0.22). In the consecutive model excluding inhibition and fingertip tapping, the only significant difference emerged for theory of mind (*F*_1,26_ = 4.80; *p* = 0.037; *n*^*2*^_*p*_ = 0.16, 95% CI 0.00–0.39).

**Fig. 6:**
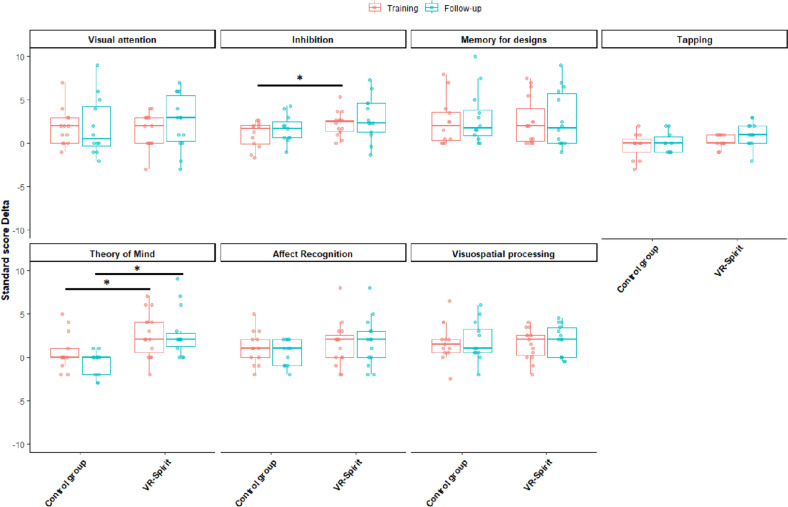
Training and follow-up effect Delta on the neuropsychological outcomes in the two groups. Asterisk lines represent significant comparisons

For the follow-up delta effect, the analysis indicated a significant group effect (*F*_7,15_ = 4.24; *p* = 0.009; *n*^*2*^_*p*_ = 0.66, 95% CI 0.08–0.72), which was specifically detected for theory of mind (*F*_1,21_ = 14.89; *p* < 0.001; *n*^*2*^_*p*_ = 0.41, 95% CI 0.09–0.62), whereas all other comparisons were non-significant (all *F* < 4.08; all *p* > 0.056). When excluding inhibition and fingertip tapping, the difference for theory of mind was still the only significant univariate result (*F*_1,24_ = 14.83; *p* < 0.001; *n*^*2*^_*p*_ = 0.38, 95% CI 0.09–0.59) (Fig. 6).

With regard to the CBCL results, no significant differences emerged (all *F* < 3.20; all *p* > 0.088), suggesting that neither the experimental nor the control intervention had an impact on emotional-behavioural problems.

### Feasibility and Acceptability

**Fig. 7:**
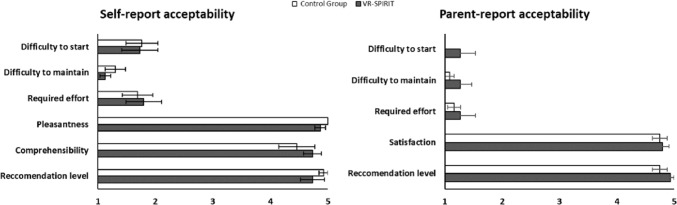
Patients and parents’ ratings of acceptability of the two interventions

As concerns feasibility, none interrupted participating in either intervention, and only one participant assigned to the VR-Spirit did not complete a session due to a sickness episode not related to the training. Moreover, the two interventions were considered similarly acceptable by both patients and their parents (all *Z* adjusted <|1.35|, all *p* > 0.17), with low scores (median for both the groups = 1) in difficulties to start, difficulties to continue the training, and in required effort, while high scores (median for both the groups = 5) were recorded for pleasantness, comprehensibility, recommendation level, and satisfaction (Fig. 7).

## Discussion

This randomised, active controlled trial tested the efficacy, feasibility and acceptability of the VR-Spirit, a VR intervention specifically designed to target social prediction abilities in patients with congenital cerebellar malformations, compared to a VR motor rehabilitation training not involving social agents. The experimental group showed a significant increase, compared to the control participants, on the prediction score of a VR social prediction task, with very large effect size. Moreover, at least at the follow-up, the VR-Spirit yielded an improvement in the use of contextual predictions in a computer-based action prediction task, with a large effect size; at individual level, a wide part of the experimental group showed an increase higher than the minimal clinically relevant change. Importantly, these effects were generalized, with a large effect size, to secondary neuropsychological outcomes, specifically theory of mind and, only for the training effect delta, inhibition. No differences between the interventions were detected on parent-reported emotional-behavioural problems. Lastly, both interventions showed high feasibility and acceptability, confirming the potential of VR as a cognitive rehabilitation tool. These results converge in sustaining future applications of VR-Spirit in different contexts and with other neurodevelopmental disorders affecting social perception.

VR-Spirit demonstrated to be effective in boosting social prediction of the avatars’ intentions, even when adopting a different scenario. Indeed, participants who completed VR-Spirit learned to predict the motor intentions of another person when choosing which direction to move in a social context. The improvement in social prediction was detectable also, at least partially, in the action prediction task. This task was previously used to investigate implicit predictive processing of social information in healthy and clinical populations (Amoruso et al., 2019; Bianco et al., 2020), and it showed to be sensitive to alterations of cerebellar functionality due to either acquired or congenital disorders (Butti et al., 2020b; Urgesi et al., 2021), as well as to the use of non-invasive brain stimulation on the cerebellum (Oldrati et al., 2021). It is worth noting that the analysis on the delta of the beta index yielded only marginally significant results, probably due to the limited sample size and consequent lack of statistical power, since we failed to reach the desired sample size according to the *a-priori* power analysis, which was conducted on the basis of this outcome (Butti et al., 2020a). Nevertheless, when directly comparing the beta index (and not the delta) at the follow-up assessment, the experimental group showed a greater reliance on contextual priors for action prediction than the control group, and this difference had a large effect size. As mentioned above, the non-significant result at the post-training assessment may depend on the limited sample size. Also, the repetition of the action prediction task in a short time may have yielded a partial improvement even in the control group, blurring between-group differences. Data reported in Table 2 and in Fig. 4 and 5 suggest that the VR-Spirit improved the beta index at T2, even though this effect did not reach statistical significance. Furthermore, at individual level, both after training and follow-up, an improvement of the beta index higher than the expected minimal clinically relevant change was detected in a wide part of the sample assigned to the VR-Spirit, but not in those assigned to the control training. Namely, participants who attended the VR-Spirit improved their ability in understanding the motor intentions of another person during the observation of simple every-day life actions.

Even more importantly, the clinical impact of the training was further corroborated by the effects on social perception, specifically on theory of mind. In fact, after the training and even at the follow-up assessment, the experimental group showed a significantly greater improvement in the theory of mind subtest compared to the control group. This means that participants who completed the VR-Spirit showed enhancements in understanding intentions, beliefs, and emotions of another person trough verbal descriptions and pictures representing different social contexts. This result ruled out the confounding effect due to the repeated administration of the same tests, confirming the specificity of the VR-Spirit in targeting the social perception domain.

Extending the preliminary evidence reported in a previous article (Urgesi et al., 2021), these findings suggest that training participants to learn explicit context-behaviour associations may improve social predictions in patients with cerebellar malformations at multiple levels, from the lower-level, implicit processing involved in action perception, to the higher-level, conceptual inferences supported by the cerebellum during the attribution of others’ mental states (Clausi et al., 2019). For the former, it is important to consider the non-significant difference between groups in the familiarisation phase of the action prediction task. Indeed, both groups were equally exposed to the implicit probabilistic associations, but only the VR-Spirit improved the use of contextual priors during the testing phase, where it was crucial to rely on predictive priors to overcome kinematics ambiguity (Frith & Frith, 2012). In this vein, the experimental training might have enhanced the matching between external, sensory information, in this case kinematics, and the internal, contextual representation formed during the familiarization, resulting into a better and timely prediction of other’s motor intentions (Molinari & Masciullo, 2019). At a higher level of social processing, the use of explicit reflective processes to understand contextualized behavioural regularities during the VR task might have trained participants to form conceptual models about others’ mental states, leading to better attributions of intentions, beliefs, and emotions in the theory of mind NEPSY-II subtest (Koster-Hale & Saxe, 2013).

The hypothesis that the VR-Spirit might be effective through the enhancement of reflective, metacognitive processes, is corroborated by the specific improvement shown by the experimental group, at least at the after-training assessment, in the inhibition subtest. Indeed, the VR-Spirit required participants to inhibit impulsive responses, which would result in random strategies like merely following the avatar’s last choice. Participants were exercised to recognize the avatars, to retrieve information on their preferences, to select the appropriate path, and to switch predictive models between avatars and different sessions. These accounts on how VR-Spirit improved social prediction and theory of mind abilities are in accordance with recent recommendations on rehabilitation of CCAS, which indicate that prompting patients to be aware of their deficits might enhance explicit reflective processes that act as an “external cerebellum”, compensating alterations of automatic, implicit cerebellar functions (Argyropoulos et al., 2020; van Dun et al., 2018).

Neither the VR-Spirit nor the active control intervention affected the presence of emotional-behavioural problems. Thus, the effects of the VR-Spirit generalized to theory of mind, but not to emotional-behavioural disorders. On one hand, an increase in neuropsychological skills does not imply the same improvement in the behavioural domain, as they represent different dimensions of psychological functioning. For instance, a child may have adequate social perception skills but appear as withdrawn or anxious, or vice versa. In fact, better abilities to process contextual cues in social contexts may increase behavioural problems, as the emergence and persistence of problem behaviours has communicative functions that are often mediated by social cues (Newcomb & Hagopian, 2018). On the other hand, despite their predominant use as behavioural outcomes, parent-report measures, as the one adopted here, have been mainly developed for healthy populations, and were not designed to detect changes over time, thus showing limited reliability and responsiveness to change in neurodevelopmental disorders (Budimirovic et al., 2017; Hanratty et al., 2015).

In terms of feasibility, only a family declined to participate before group allocation, and only one session of the VR-Spirit was interrupted due to a sickness episode unrelated to the training. Moreover, both participants and their parents reported optimal ratings at the acceptability questionnaire. These results sustain that, although it is based on task repetition, the VR-Spirit is feasible and acceptable for patients with a wide range of cognitive functions, from moderate intellectual disability to (above) average intellectual functioning. Notably, the two interventions were comparable on the acceptability scores, confirming the potential of VR as a cognitive rehabilitation tool in populations with neurodevelopmental disorders (Nossa et al., 2022; Tieri et al., 2018). The high ratings attributed by parents might depend on the lack of specific interventions for children and young adults with congenital cerebellar malformations (Argyropoulos et al., 2020; Romaniello et al., 2022), so that they were highly motivated and enthusiastic to have their son or daughter participate in either VR training. Also, the high ratings in recommendation level and satisfaction provide preliminary indications on the social validity of the VR-Spirit (Strain et al., 2012; Wolf, 1978). Moreover, the comparable ratings on the acceptability questionnaires, along with the fact that both trainings were delivered in the same VR laboratory, exclude that the differences between groups in the primary and secondary outcomes may be due to a higher placebo effect for the VR-Spirit or to motivational aspects.

Limitations must be acknowledged when discussing results of the study. First, the spread of the Covid-19 pandemic prevented the pre-estimated sample size from being reached, undermining the analysis power for the primary outcome. Nevertheless, the analyses, which were previously defined and published (Butti et al., 2020a), yielded convergent results on the efficacy of the VR-Spirit. Moreover, despite a high variability of intellectual functioning in our sample, representative of populations with cerebellar malformations (Bulgheroni et al., 2016; Summers et al., 2017; Tavano et al., 2007), the limited sample size prevented testing differential effects of the training in relation to cognitive level. Moreover, even though all participants presented with cerebellar malformations, diverse patterns of malformations (e.g., the molar tooth sign associated with Joubert syndrome) might differentially affect the neuropsychological functioning and thus social prediction abilities (Butti et al., 2023; Tavano & Borgatti, 2010), influencing also the effects of the rehabilitative training. Lastly, the neuropsychologist conducting the assessment was not blind to the intervention; however, group allocation was determined on the basis of the order of hospital admission, which was organized by an administrative staff who was blind to the study aims and procedures.

In conclusion, these findings confirm that it is possible to develop condition-specific rehabilitative trainings on the basis of the predictive processing exerted by the cerebellum (Bhanpuri et al., 2014). The VR-Spirit demonstrated to generalize its effects to theory of mind abilities, and it might be thus extended to other neurodevelopmental disorders that present social perception deficits and alterations of predictive processing, such as ASD (Amoruso et al., 2019; Pellicano & Burr, 2012) and Williams Syndrome (Sparaci et al., 2014). By boosting the ability to predict other’s motor intentions in a virtual environment, the VR-Spirit may be effective in improving navigation skills, which are often impaired in these neurodevelopmental disorders and in conditions of early brain damage (Broadbent et al., 2014; Nossa et al., 2022; Pavlova et al., 2007). Lastly, the Covid-19 pandemic has urged the medical and scientific community to develop new strategies and tools for remote assessment and rehabilitation. Tele-rehabilitation indeed provides an opportunity to keep fragile individuals safe, as well as to improve outreach and ensure compliance with a rehabilitation plan. This is particularly true for those patients that require long-term care and follow-up, as for cerebellar patients. Although the need of further research on this topic, the application of VR may offer new and promising possibilities for tele-rehabilitation of social skills in populations with intellectual and developmental disabilities (Montoya-Rodríguez et al., 2023). The use of VR for supporting social skills in neurodevelopmental disorders may also shorten acquisition times than traditional interventions (Frolli et al., 2022). Considering VR-based technologies, immersive VR viewers have proven to be able to foster more efficient functional performance and cognitive improvement than non-immersive VR (Chatterjee et al., 2022; Cikajlo & Peterlin Potisk, 2019). Notably, these technologies are affordable and more and more available in everyday life because of the spreading of immersive VR for gaming. Accordingly, the VR-Spirit has been recently adapted to commercial VR-viewers, becoming accessible for a larger number of patients, and it will be tested for tele-rehabilitation (Malerba et al., 2023).

## Supplementary Information

Below is the link to the electronic supplementary material.

Supplementary file1 (DOCX 24 KB)

## Data Availability

The study protocol and summary of basic results are registered and published: https://www.isrctn.com/ISRCTN22332873. Data analysed in this study are available through a public repository at the following link: https://zenodo.org/record/8192485.
