# Supplementary material for: Feasibility and Efficacy of a Virtual Reality Social Prediction Training in Children and Young Adults with Congenital Cerebellar Malformations
Source: J Autism Dev Disord. 2024 May 3;55(7):2463–79. doi: 10.1007/s10803-024-06349-8 (PMC12167251; doi:10.1007/s10803-024-06349-8)
Supplement: Supplementary file 1 — Supplementary file1 (DOCX 24 KB) [file 10803_2024_6349_MOESM1_ESM.docx]

**Supplementary table 1. Participants assigned to each stratification block according to the allocation sequences.** Blue and red numbers indicate participants allocated to the VR-Spirit (S) and the control training (C), respectively**.** Please note that C13 was originally assigned to the block with IQ > 80 according to previous cognitive assessments. Unnumbered elements represent allocations that were not fulfilled due to recruitment issues.

|  |  | **Age (in years)** | |
| --- | --- | --- | --- |
|  | | **7-12.9** | **≥13.0** |
| **IQ** | **46-60** | **C3-C5-S9-S10-C8-C10-S13-**S | **S4-C9-S11-C11-S14-C12-**S-C |
|  | **61-80** | **S1-C4-C6-S8-**S**-C13-**C-S | **S2-S5-**C-C-S-S-C-C |
|  | **>80** | **C1-S3-C2-S12-**C**-S15-**C-S | **C7-S6-S7-**C-C-S-S-C |

**Supplementary table 2. Clinical information of participants enrolled in the VR-Spirit.**

| **ID** | **Sex** | **Age (years)** | **FSIQ** | **Cerebellar malformation** | **Other brain abnormalities** | **Syndrome or genetic diagnosis** | **Speech** | **School** | **Clinical features** |
| --- | --- | --- | --- | --- | --- | --- | --- | --- | --- |
| S1 | M | 12.9 | 77 | Vermis hypoplasia |  | Unknown | Dysarthria - words and sentences | Differentiated and reduced school programme | Diffuse hypotonia, clumsiness |
| S2 | M | 23.8 | 64 | Molar tooth | Anomalous venous drainage in right frontal areas | Joubert syndrome (KIAA0586 gene mutation) | Preserved | Completed a technical school with special education | Diffuse hypotonia, clumsiness, ataxia, dysmetria |
| S3 | M | 9.4 | 81 | Left hemisphere hypoplasia, right hemisphere dysplasia | Pons hypoplasia | Unknown | Preserved | Differentiated school programme | Diffuse hypotonia, clumsiness, dysmetria, learning difficulties |
| S4 | M | 14.3 | 48 | Vermis and upper bilateral hemispheres atrophy |  | ITPR1 gene mutation | Dysarthria - words and sentences | Differentiated and reduced school programme | Diffuse hypotonia, clumsiness, ataxia, dysmetria, tremors, nystagmus |
| S5 | F | 14.7 | 73 | Vermis hypoplasia | Complete agenesis of corpus callosum, periventricular nodular heterotopia | Unknown | Preserved | Differentiated school programme | Diffuse hypotonia, clumsiness, obesity, learning difficulties |
| S6 | M | 13.6 | 137 | Mild vermis and bilateral hemispheres hypoplasia | Megacisterna magna | Unknown | Preserved | Normal school programme | Spastic dyparesis, toe walking |
| S7 | F | 13 | 90 | Romboencephalosynapsis |  | Unknown | Preserved | Differentiated school programme | Clumsiness, bilateral neurosensorial hypoacusia |
| S8 | F | 8.1 | 76 (PRI) | Vermis hypoplasia |  | Unknown | Dysarthria - Few words | Differentiated and reduced school programme | Clumsiness |
| S9 | M | 11.8 | 52 | Vermis and bilateral hemispheres hypoplasia |  | Unknown | Dysarthria - words and sentences | Differentiated and reduced school programme | Diffuse hypotonia, clumsiness, dysmetria, bilateral neurosensorial hypoacusia,ataxia |
| S10 | M | 12.9 | 46 | Molar tooth | Anterior mesencephalic cap dysplasia | Joubert syndrome (TMEM67 gene mutation) | Dysarthria - Absent | Differentiated and reduced school programme | Diffuse hypotonia, clumsiness, ataxia, dysmetria, scoliosis, oculomotor apraxia, visual impairment, strabismus, nystagmus |
| S11 | M | 15.1 | 46 | Vermis hypoplasia | Corpus callosum hypoplasia | Unknown | Dysarthria - Few words | Differentiated and reduced school programme | Diffuse hypotonia, clumsiness, subclinical hypothyroidism, esotropia(left eye), obesity, facial dysmorphisms |
| S12 | M | 10.1 | 84 | Hypoplasia, elevation, and upward rotation of the cerebellar vermis (Dandy-walker malformation) | Cystic dilatation of the IV ventricle, Corpus callosum dysmorphisms | Unknown | Preserved | Differentiated school programme | Macrocrania, facial dysmorphisms, clumsiness, learning difficulties |
| S13 | M | 12.1 | 50 | Vermis hypoplasia | Corpus callosum hypoplasia | Unknown | Dysarthria - words and sentences | Differentiated and reduced school programme | Diffuse hypotonia, clumsiness, obesity, facial dysmorphisms |
| S14 | M | 17.6 | 46 | Molar tooth | Corpus callosum dysmorphisms | Joubert syndrome (Unknown genetics) | Dysarthria - words and sentences | Differentiated and reduced school programme | Diffuse hypotonia, clumsiness, ataxia, oculomotor apraxia, nystagmus, isolated GH deficiency, G6PD deficiency |
| S15 | F | 11.0 | 110 | Cerebellar folia dysplasia | Complete agenesis of corpus callosum | Unknown | Preserved | Differentiated school programme | Learning difficulties |

**Supplementary table 3. Clinical information of participants enrolled in the active control group.**

| **ID** | **Sex** | **Age (years)** | **FSIQ** | **Cerebellar malformation** | **Other brain abnormalities** | **Syndrome or genetic diagnosis** | **Speech** | **School** | **Clinical features** |
| --- | --- | --- | --- | --- | --- | --- | --- | --- | --- |
| C1 | M | 10.3 | 120 | Mild vermis hypoplasia | Megacisterna magna, Hydrocephalus | Unknown | Preserved | Normal school programme | Diffuse hypotonia, clumsiness |
| C2 | F | 12.1 | 86 | Molar tooth, right hemisphere dysplasia |  | Joubert syndrome (CEP290 gene mutation) | Preserved | Differentiated school programme | Diffuse hypotonia, obesity, retinal dystrophy, learning difficulties |
| C3 | M | 8.6 | 57 | Molar tooth | Anterior mesencephalic cap dysplasia | Joubert syndrome (Unknown genetics) | Dysarthria - words and sentences | Differentiated and reduced school programme | Diffuse hypotonia, clumsines , ataxia, dysmetria, facial dysmorphisms, hands and feet post-axial polydactyly |
| C4 | M | 11.7 | 76 | Mild vermis and hemisphere hypoplasia (left> right) |  | Unknown | Preserved | Differentiated school programme | Diffuse hypotonia, clumsiness, facial dysmorphisms, bilateral congenital pes tortus |
| C5 | M | 7.4 | 52 | Mild vermis and bilateral hemispheres hypoplasia | Medulla oblongata and pons dysmorphisms | Unknown | Dysarthria - words and sentences | Differentiated and reduced school programme | Diffuse hypotonia, clumsiness |
| C6 | M | 10.1 | 68 | Mild vermis hypoplasia | Pons hypoplasia, basal ganglia and corpus callosum dysmorphisms | Unknown | Preserved | Differentiated school programme | Clumsiness |
| C7 | M | 25.6 | 87 | Molar tooth | Hamartomatous formation in the floor of the III ventricle | Unknown | Dysarthria - words and sentences | Completed high scool with special education | Clumsiness |
| C8 | F | 9.6 | 48 | Molar tooth | Irregular medulla-pontine junction, olfactory bulb hypoplasia, small heterotopic nodules | Joubert syndrome (AHI1 gene mutation) | Dysarthria - words and sentences | Differentiated and reduced school programme | Diffuse hypotonia, dysmetria, hypodiadococinesia, oculomotor apraxia, nystagmus |
| C9 | M | 17.2 | 46 (PRI) | Molar tooth | Anterior mesencephalic cap dysplasia | Joubert syndrome (TMEM67 gene mutation) | Dysarthria - Absent | Differentiated and reduced school programme | Diffuse hypotonia, clumsiness, ataxia, dysmetria, scoliosis, oculomotor apraxia, visual impairment, strabismus, nystagmus, ptosis right eye |
| C10 | M | 11.5 | 58 | Molar tooth | Corpus callosum dysmorphisms | Joubert syndrome (CEP104 gene mutation) | Dysarthria - words and sentences | Differentiated and reduced school programme | Diffuse hypotonia, clumsiness, dysmetria,ataxia, nystagmus, epilepsy, obesity |
| C11 | M | 14.8 | 51 | Vermis hypoplasia | Bilateral perisylvian polymicrogyria, aberrant supracallosal longitudinal bundle | Unknown | Preserved | Differentiated and reduced school programme | Right eye exotropia |
| C12 | M | 18.9 | 55 | Molar tooth |  | Joubert syndrome (Unknown genetics) | Dysarthria - words and sentences | Differentiated and reduced school programme | Diffuse hypotonia, dysmetria, tremors, dysdiadochokinesis, ataxia, oculomotor apraxia, nystagmus |
| C13 | F | 8.4 | 65 | Molar tooth |  | Joubert syndrome (KIAA0586 gene mutation) | Preserved | Differentiated school programme | Diffuse hypotonia, clumsiness, ataxia |
